# Zebrafish *eda* and *edar* Mutants Reveal Conserved and Ancestral Roles of Ectodysplasin Signaling in Vertebrates

**DOI:** 10.1371/journal.pgen.1000206

**Published:** 2008-10-03

**Authors:** Matthew P. Harris, Nicolas Rohner, Heinz Schwarz, Simon Perathoner, Peter Konstantinidis, Christiane Nüsslein-Volhard

**Affiliations:** Max Planck Institute for Developmental Biology, Tübingen, Germany; University of Helsinki, Finland

## Abstract

The genetic basis of the development and variation of adult form of vertebrates is not well understood. To address this problem, we performed a mutant screen to identify genes essential for the formation of adult skeletal structures of the zebrafish. Here, we describe the phenotypic and molecular characterization of a set of mutants showing loss of adult structures of the dermal skeleton, such as the rays of the fins and the scales, as well as the pharyngeal teeth. The mutations represent adult-viable, loss of function alleles in the *ectodysplasin* (*eda*) and *ectodysplasin receptor* (*edar*) genes. These genes are frequently mutated in the human hereditary disease hypohidrotic ectodermal dysplasia (HED; OMIM 224900, 305100) that affects the development of integumentary appendages such as hair and teeth. We find mutations in zebrafish *edar* that affect similar residues as mutated in human cases of HED and show similar phenotypic consequences. *eda* and *edar* are not required for early zebrafish development, but are rather specific for the development of adult skeletal and dental structures. We find that the defects of the fins and scales are due to the role of Eda signaling in organizing epidermal cells into discrete signaling centers of the scale epidermal placode and fin fold. Our genetic analysis demonstrates dose-sensitive and organ-specific response to alteration in levels of Eda signaling. In addition, we show substantial buffering of the effect of loss of *edar* function in different genetic backgrounds, suggesting canalization of this developmental system. We uncover a previously unknown role of Eda signaling in teleosts and show conservation of the developmental mechanisms involved in the formation and variation of both integumentary appendages and limbs. Lastly, our findings point to the utility of adult genetic screens in the zebrafish in identifying essential developmental processes involved in human disease and in morphological evolution.

## Author Summary

A major goal of the study of developmental genetics is to understand the genes and developmental mechanisms underlying the formation of organismal complexity and diversity. Here, we focus on genes controlling postembryonic development and describe mutations in genes of the ectodysplasin (Eda) pathway in regulating the formation of the scales, skull, fins, and teeth. Mutations in genes of this signaling pathway are common in humans with defects in ectodermal structures such as hair, glands, and teeth. We show that the similar phenotypes of loss of Eda signaling in fish and human are due to a conserved early developmental stage in the development of mammalian hair and fish scales; subsequent development of these two structures diverge. Our findings show that the Eda signaling pathway has an ancestral role in regulating the developmental interactions involved in patterning and growth of the dermal skeleton of fish. Recent work has shown that these genes are associated with morphological variation between humans and evolution within fish populations, suggesting that alteration in the function of these genes permits viable morphological change. Our data support the value of forward genetic studies on postembryonic development to reveal the genetic and developmental basis of both human disease and morphological evolution.

## Introduction

The genetic and developmental basis of the formation of organismal shape and form is a long-standing question in biology. The analysis of mutations has been essential in identifying the genes and regulatory networks underlying development. However, while the genetic basis of embryonic development has been extensively studied by systematic mutagenesis screens, we know little of the genes involved in the development of adult morphology. Yet, it is the heritable variation in adult form that natural selection primarily acts on during evolution. In order to understand the basis of variation, we need to know more about the genetic control of the development of adult form: which genes are involved, what are their function, and when are they required in development [1],[2]. To identify genes important for development of adult structures, we initiated a large-scale mutagenesis screen in zebrafish and scored for mutants affected in the shape and pattern of adult structures. We isolated only adult viable mutants, therefore we selected for genes that have an increased probability to be involved in morphological change during evolution. Identification of zebrafish genes homologous to human genes associated with disease that arise during postnatal development into adulthood is also likely in this screen.

We focused on mutants that exhibit defects in the dermal skeleton of the adult zebrafish. The dermal skeleton encompasses the external form of the adult fish. The most prominent dermal skeletal elements are the dermocranium of the skull and lateral bones of the opercular series, the scales, and the fin rays (or lepidotrichia). Additionally, the teeth and gill rakers (bones that support the gills in teleosts) are elements of the dermal skeleton [3],[4]. Unlike the ossification process that occurs during endochondrial bone development in which organic matrix is deposited by osteoblasts over a chondrogenic scaffold, dermal skeletal elements originate as direct mineralization of a collagenous matrix deposited by dermal fibroblasts. This process occurs in close association with the epidermis. The initiation and patterning of dermal elements are thought to be similar to epidermal appendages (e.g. hairs and feathers) and is controlled by reciprocal signaling between an epithelium and mesenchyme (see [5],[6]). Importantly, in zebrafish, as in most teleosts, the majority of dermal skeletal elements are not formed during larval development, rather through juvenile metamorphosis and development of the adult pattern. Those that begin to form in late larval development such as the teeth and gill rakers, do not fully attain their shape and pattern until juvenile metamorphosis.

Variations in the shape of dermal skeletal elements of the fins, scales, cranium, and teeth play a significant role in adaptations of fish populations to new environments (e.g. dermal plate development and stickleback radiation [7]). Additionally, integumentary appendages, such as hair and feathers, have been essential and defining traits of vertebrate classes. Early vertebrates, the conodonts, ostracoderms and placoderms, possessed a pronounced dermal skeleton, often in the absence of an ossified axial skeleton [8]. Through vertebrate evolution from fish to tetrapods, dermal structures such as lateral bones of the opercular series, scales, dermal plates and fin rays were either reduced or lost. This evolutionary transition was paralleled with the elaboration of the cartilaginous endoskeleton of the limbs and the evolution of specialized keratinized appendages of the integument such as epidermal scales, feathers and hairs. In contrast, the diversity of form in extant bony fishes involves modification in size, shape and number of the scales/dermal plates, fin rays, cranial dermal bones and teeth.

Here, we describe a collection of mutants that have shared defects in the formation of the dermal skeletal elements of the skull, fins, scales and teeth of the adult zebrafish. The mutations disrupt the genes *ectodysplasin* (*eda)* and *edar* encoding the *eda* receptor. In mammals the EDA signaling pathway is involved in hair and teeth formation [9] and mutations affecting this pathway cause the human hereditary disease hypohidrotic ectodermal dysplasia (HED). Loss of Eda signaling in the zebrafish causes a spectrum of phenotypes corresponding to those described for HED in humans, and therefore the zebrafish mutants may serve as a genetic model of this disease. We describe the requirement of Eda signaling in the zebrafish epidermis for the formation of a structure resembling an epidermal placode seen in the early development of other vertebrate integumentary appendages. The mutations also result in defects of skeletal elements unique to fish and suggest an ancestral role of Eda signaling in the formation and patterning of the dermal skeleton. Lastly, whereas loss of function of Eda signaling causes a severe phenotype, the expressivity of dominant alleles is sensitive to background modifiers that buffer the phenotypic consequences of loss of Eda signaling. Additionally, we find that the response to reduction of Eda signaling is dose sensitive and organ specific. We suggest that such alleles may provide a basis for morphological variation in evolution.

## Results

### *finless* and *Nackt* Mutants Exhibit Defects in the Development of the Dermal Skeleton

**Figure 1:**
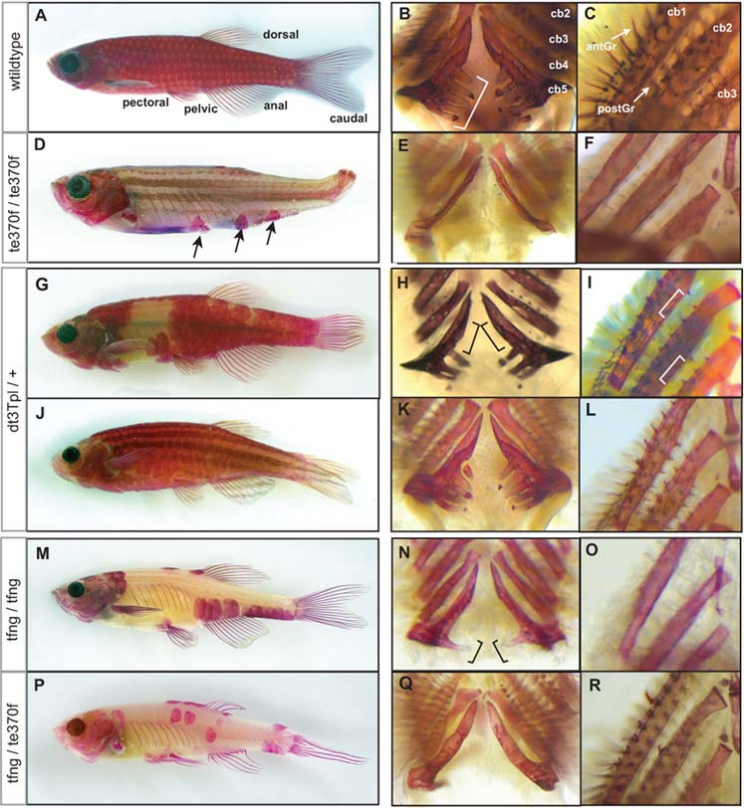
The formation of the adult dermal skeleton and pharyngeal teeth is affected in *fls* mutant zebrafish. A) Alizarin red-stained wild type adult zebrafish shows staining of the scales, fin rays, dermal bones of the skull as well as the pharyngeal teeth along ceratobranchial 5 (bracket, B) and (C) gill rakers along both the anterior (antGr) and posterior edge (postGr) of non-teeth bearing ceratobranchials. D) *fls* ^te370f^ shows loss of dermal skeletal structures of the fin rays, scales and alteration in the shape of the skull. Additionally, *fls* ^te370f^ shows loss of pharyngeal teeth (E) and gill rakers (F). G–I) The Topless allele (*fls^dt3Tpl^*) shows a dominant effect on scalation and tooth/gill raker formation while not affecting lepidotrichial growth. J–L) Expressivity of *fls* ^dt3Tpl^ is sensitive to a modifier in the Tü strain leading to a “weak” *fls* ^dt3Tpl^ phenotype; *fls* ^dt3Tpl^ homozygotes were phenotypically identical to *fls* ^te370f^ (not shown). The fang allele of *fls*, *fls* ^tfang^, isolated in a non-complementation screen with *fls* ^te370f^, shows no effect on fin development while exhibiting partial loss of scales (M), teeth (N), and gill rakers (O). Transallelic tfang/te370f zebrafish exhibit an intermediate phenotype between homozygous *fls*(te370f) and *fls*(tfang) (P–R).

In a mutagenesis screen for mutations affecting adult zebrafish structures, we identified three mutants that showed nearly identical defects in the formation of scales, lepidotrichia, and shape of the skull of homozygous fish. These mutants fell into two complementation groups. The first is allelic to the *finless (fls* ^te370f^ *)* mutant that was previously isolated in the background of the Tübingen wild type stock (Tü) on the basis of the loss of fins in adults [10]. We isolated two new alleles of *fls* in the screen and found another in the background of the *TLF* wild type stock. The majority of the *fls* alleles isolated are recessive and have a strong phenotype (see below). However, the *fls* ^dt3Tpl^ allele is dominant with a partial scale loss phenotype in heterozygotes (Figure 1G, J). One further *fls* allele was isolated in a screen for mutations that failed to complement the *fls* ^te370f^ mutant (Figure 1M). We named this allele *fang* (*fls* ^tfng^) after its unique dental phenotype in homozygotes of having only one tooth on the fifth ceratobranchial (Figure 1N). The *fls* ^tfng^ allele shows no effect on fin development and has a slight increase in the number of scales than the other *fls* alleles isolated.

**Figure 2:**
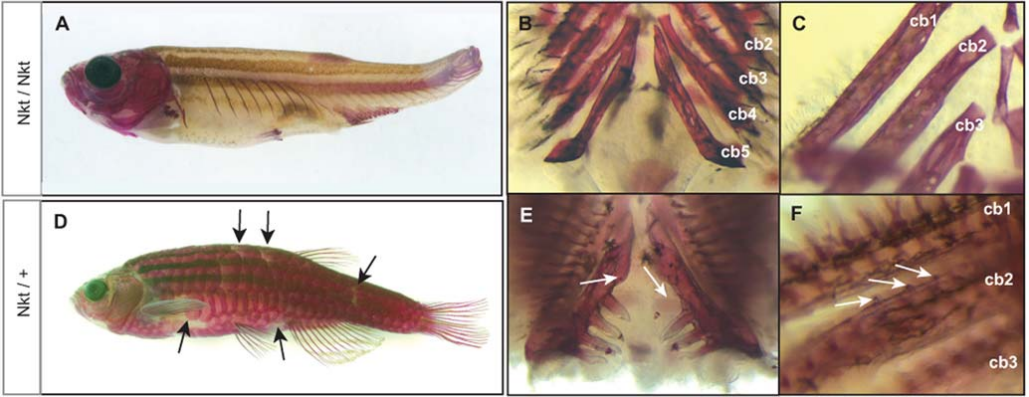
The dominant gene *Nkt* is phenotypically similar, however complements *fls* mutants. *Nkt* homozygotes show complete loss of scales, teeth and gill rakers resembling the *fls* phenotype (A–C). Heterozygous *Nkt* zebrafish show an intermediate phenotype of scale loss and patterning defect (arrows) while no effect on fin development is seen (D). Heterozygous *Nkt* also show a dominant effect on the number of teeth (arrows, E) and gill rakers (F), showing deficiencies along the posterior branchial arches and formation of rudimentary rakers along ceratobranchial 1 and 2 (arrows, F). *Cb1-5*, ceratobranchial bones.

The second complementation group was comprised of a single gene, which we called *Nackt* (*Nkt*). This allele is dominant causing a slight defect in the patterning and shape of scales as heterozygotes (Figure 2D). The homozygous phenotype is more severe than that of strong *fls* alleles (Figure 2A).

Phenotypic defects of *fls* and *Nkt* mutants become apparent in juvenile fish; as larvae, homozygous *fls* and *Nkt* mutants are visibly unaffected. Homozygous adults are viable, and of normal size. With the exception of the *fls* ^tfng^ allele, the lepidotrichia that form during juvenile metamorphosis are defective, leading to fin loss in the adult (Figure 1D, 2A). The dermal bones of the pectoral girdle are present and patterned appropriately in both the *fl*s and *Nkt* mutants. By close examination of the visceral skeleton we found that neither the pharyngeal teeth, nor the bony substrates of the gills, the gill rakers, are formed (Figure 1E and F; Figure 2B, C). In addition, scales are largely absent with infrequent formation of inappropriately shaped scales near the dorsal, anal and pectoral fins (Figure 1D, 2A). *Nkt* homozygous fish exhibit more severe defects in the formation of the dermal skeleton than *fls* alleles in the extent of lepidotrichial growth and number of residual scales formed (compare Figure 1D and Figure 2A). The skull of mutants has a normal appearance with all the bones being present, although the size, shape and relative proportion of the various bones differ compared to wild type individuals (Figure S1); no change in cranial shape was apparent in larvae.

*Nkt* heterozygous fish exhibit a dominant phenotype as they lack several scales on the flank and those present at the flank are elongated dorso-ventrally. The number of teeth and gill rakers is reduced, however lepidotrichia formation and growth of the fins are not affected (Figure 2D–F). The skulls of *Nkt* heterozygotes do not show increased size, but retain altered shape and proportion as seen in homozygotes (Figure S1).

### The Topless Allele of *fls* Uncovers Background Specific Modulation of *fls* Expressivity

We isolated a dominant allele of *fls* that exhibits a distinct phenotype in heterozygotes that we named *Topless* (*fls* ^dt3Tpl^). Heterozygous *fls* ^dt3Tpl^ have a reduction in the number of scales, teeth and gill rakers, but show little to no effect on lepidotrichia development (Figure 1G–I). Mutant *fls* ^dt3Tpl^ fish exhibit the strong *fls* phenotype when homozygous or heterozygous with other *fls* alleles. Similar to *Nkt*, *fls* ^dt3Tpl^ exhibits a dominant effect on skull shape as well (Figure S1).

The expressivity of the dominant *fls* ^dt3Tpl^ phenotype depends on the genetic background. Fish heterozygous for *fls* ^dt3Tpl^ exhibited either a “strong” or a “weak” phenotype in the Tü background (Figure 1G and J, respectively). The “strong” phenotype shows loss of scales regionally in the midflank, loss of medial pharyngeal teeth along the fifth ceratobranchial and loss of posterior gill rakers of the anterior arches (Figure 1G–I). In contrast, the “weak” phenotype displays only subtle variation in scale patterning and no effect on the teeth or gill rakers could be detected (Figure 1J–L). The segregation pattern of the two phenotypic classes of *fls* ^dt3Tpl^ suggests the presence of separate, unlinked, modifier loci in the Tü background affecting the number of scales (Table 1 and data not shown). Additionally, we found that the *fls* ^dt3Tpl^ “strong” phenotype was partially suppressed when crossed with the polymorphic WIK mapping strain indicating the presence of dominant modifier(s) in the WIK line (Table 1). The resulting heterozygous progeny showed reduced scale-loss compared to *fls* ^dt3Tpl^ heterozygotes in a Tü background, but had similar defects in scale shape (Table 1). Therefore, dominant modifier loci are present in the WIK strain that buffer the expressivity of the *fls* ^dt3Tpl^ dominant phenotype. None of the other *fls* alleles showed any dominance in the Tü, TLF, or WIK strains.

**Table 1:** Quantitative effect of *fls* on scale number and shape and the effect of background modifiers in *Danio rerio* strains on *fls* ^dt3Tpl^.

Phenotype/Genotype	Scale #/ stl	n	Scale DV/AP	n
		fish		scales
+/+	6.8±0.18	4	1.14±0.15	13
*fls* ^dt3Tpl^ / Tü	3.0±0.20 ##	2	1.52±0.29 #	8
*fls* ^dt3Tpl^ / Tü; *mod*	5.6±0.44 #	3	1.4±0.3 #	12
*fls* ^dt3Tpl^ / WIK	5.84±0.66 #	9	1.43±0.35 #	32
*fls* ^tfang^ / *fls^t^* ^fang^	0.97±0.50 ###	2	1.57±0.18 #	7
*fls* ^te370f^ / *fls* ^te370f^	0.41±0.39 ###	6	1.8±0.64 #	16

The total number of scales on one side of alizarin red stained adults of different genotypes were counted and measured. Counts were normalized for standard length (stl) of individual fish as shape and number of scales in the mutants may vary as a measure of size. Shape characteristics of scales were quantified by measuring three to four scales from set positions across the flank of each fish and comparing the height (dorsal-ventral; DV) to length (anterior-posterior; AP) ratios. Results are presented as sample average and standard deviation around the mean. *mod*, inferred genotype of a modifier in Tü background leading to “weak” phenotype. The numerical symbol (#) indicates significant difference compared to wild type values (students *t*, p<0.05). The different number of symbols signifies a significantly different phenotypic classes of scale development (#, ##, ###).

### Mutations in *fls* Disrupt the Ectodysplasin Receptor in Zebrafish

We identified the affected loci of the *fls* mutants by positional cloning. The *fls* ^te370f^ mutation was linked to SSLP markers on linkage group 9 (LG9). Due to similarity of the *fls* phenotype to ectodermal dysplasia phenotypes in mammals, we mapped several genes of the ectodysplasin pathway to the zebrafish radiation hybrid map to see if any of these genes were linked to *fls*. The *edar* gene is located on LG9 within the determined linkage interval for *fls* (see Methods). We cloned the full-length wild type cDNA of *edar* and found several polymorphisms in the Tü *edar* cDNA when compared to the WIK mapping strain; these polymorphisms were tightly linked with the *fls* mutation and did not show recombination in 238 meioses (Figure S2).

**Figure 3:**
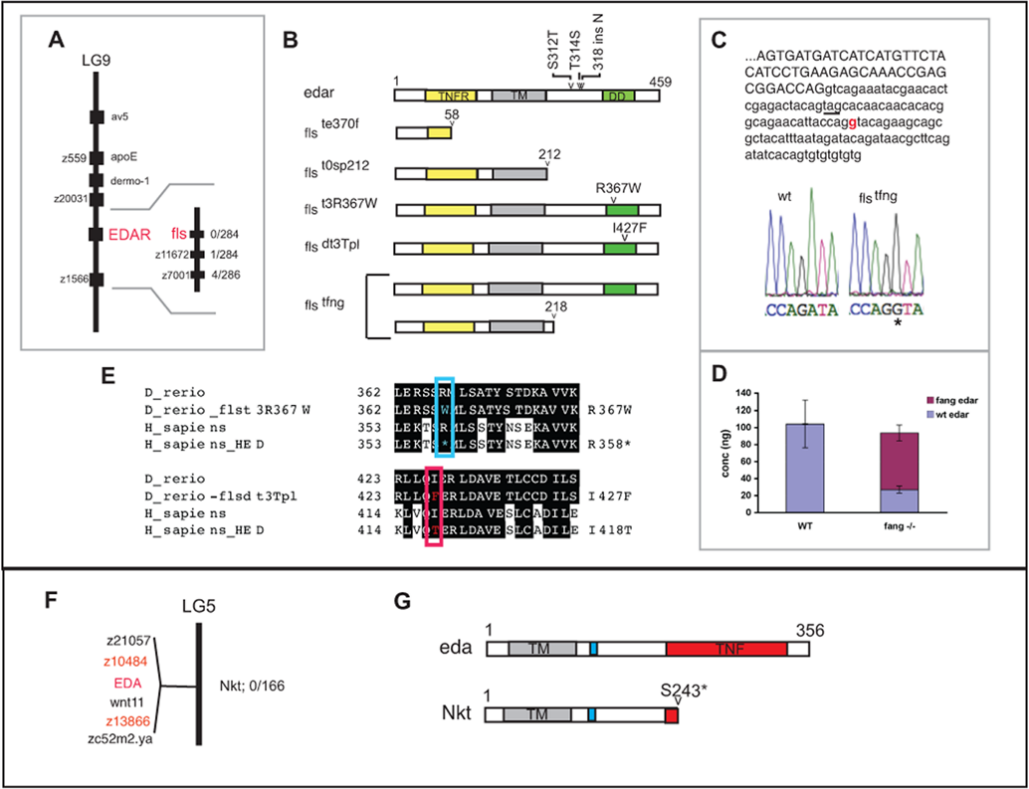
*fls* and *Nkt* are mutations in genes encoding ectodysplasin receptor (*edar*) and its ligand ectodysplasin (*eda)*. A) Mapping of *fls* using SSLP markers and placement of the *edar* gene within the candidate region on LG 9 by radiation hybrid mapping. The insert shows genetic linkage of the *fls* gene to local markers on LG 9. The numbers on the right of the insert indicate the number of recombinants seen in identified mutants per the number of meioses tested. B) Schematic of wild type Edar protein and mutant alleles. Polymorphisms seen in the WIK strain are noted above the wild type gene. Mutations that lead to premature termination are represented as truncated proteins showing the predicted residual fragment and position of the identified mutation. C) Analysis of the mutation in *fls* ^dfang^. A unique splice donor site (red) is generated leading to inclusion of additional coding sequence encoding a premature termination codon (underlined). D) Quantitative analysis of different *edar* transcript levels in *fls* ^dfang^ homozygotes compared with wildtype. E) Similarity of altered residues in *fls* ^t3R367W^and *fls* ^dt3Tpl^ with human HED shown in the death domain. The position of the mutated residues in *fls* ^t3R367W^ (blue box) and in *fls* ^dt3Tpl^ (red box) is identical to ones changed in cases of human autosomal dominant HED although the substitution is different. F) Linkage between the *Nkt* allele and *eda* on LG5. G) Schematic of wild type Eda protein and position of *Nkt* mutation. Numbers on gene diagrams represent amino acid length. *TNF*, tumor necrosis factor domain; *TNFR*, tumor necrosis factor receptor domain; *TM*, transmembrane domain; *DD*, death domain; the blue box in *Eda* is the furin binding site.

The *edar* gene encodes a transmembrane protein with similarity to tumor necrosis factor receptor (TNFR). The Edar protein contains a conserved TNFR extracellular ligand binding domain and a cytoplasmic terminal death domain essential for protein interactions with signaling adaptor complexes. The *fls* ^te370f^ mutation is an A to T transversion at a splice acceptor site, resulting in missplicing of the mRNA leading to a frame shift in translation and the generation of a premature stop codon (Figure 3B and Figure S2). This allele is a likely molecular null mutation as only a fragment of the ligand-binding domain is present while the transmembrane and cytoplasmic death domains, which are essential for function of this protein, are both absent. The spontaneous mutation *fls* ^t0sp212^ was found to have a splicing defect leading to the inclusion of intronic sequence. This is predicted to form a protein with incorrect amino acid sequence after residue 212, at the end of the transmembrane domain leading to a premature termination codon (Figure 3B, Figure S2). The two alleles generated by the ENU mutagen both have missense mutations resulting in amino acid changes in the death domain (*fls* ^t3R367W^, R367W^(C-T)^; *fls* ^dt3Tpl^, I428F ^(A-T)^). These mutations were found at identical positions as seen in familial cases of HED in humans (Figure 3B, E; [11],[12].

### The *fang* Allele Uncovers Dose and Organ Specific Sensitivity to Levels of Eda Signaling

The *fang* allele of *fls* was isolated in an allele screen for mutants that failed to complement *fls^te370f^* (Figure 1P). *fls^tfng^* homozygotes do not show any observable effect on lepidotrichia development yet have a reduction of scales and teeth/rakers as seen in other *fls* alleles (Figure 1M–O). The fang allele in *trans* to the te370f putative null allele shows an intermediate phenotype affecting lepidotrichial growth and a further reduction of teeth and scales suggesting that the *fang* allele is a hypomorph (Figure 1P–R); *fls^tfng^* heterozygotes do not show any differences compared to wild type. The shape and number of the scales in *fang* is similar to the other homozygous *fls* alleles (Table 1). Analysis of *edar* RNA from homozygous *fls* ^tfng^ showed the presence of two distinct transcripts with an additional larger isoform than seen in wildtype. Analysis of the sequence of the novel isoform showed the addition of intronic sequence leading to a premature termination codon (Figure 3C). The predicted protein would be similar to the *fls* ^t0sp212^ allele having truncation just after the transmembrane domain at amino acid 218 (Figure 3B and Figure S2). Analysis of the genomic sequence in the mutant revealed that the altered splicing is due to an A to G transition leading to the creation of a new splice donor site in the intron (Figure 3C). Given the presence of both isoforms in homozygous individuals, this novel splice site is used in addition to the normal splice junction. Using quantitative real time PCR we found that the *fang*-specific *edar* transcript represents 74% of the total pool of *edar* transcripts in homozygous mutants (Figure 3D). The dilution of wild type transcripts can explain the observed hypomorphic effect of the allele. From this unique allele of *fls*, it is clear that the phenotypic effect of loss of Eda signaling is dose dependent and that scales and teeth are more sensitive to alterations in the level of Eda signaling than fins.

### Ectodysplasin Is Mutated in the *Nackt* Mutant

EDAR and its orthologue XEDAR recognize specific EDA isoforms that vary by two amino acids [13]–[16]. The receptor-ligand complex signals though NF-κB using several adaptor proteins that are generally specific to each receptor. Together, mutations in *Edar* and *Eda* lead to the majority of cases of human HED in which the development of integumentary appendages (hairs, glands and teeth) are affected (OMIM 300451, [17]; OMIM 604095 [18]).

We reasoned that, because of the similarity in phenotype to *fls*, the *Nkt* gene could be *eda*, encoding the ligand for Edar. We isolated the entire coding region for zebrafish *eda* by RACE (Figure S3). The *eda* transcript from the *Nkt* mutant shows a precocious stop codon predicting a truncation of the protein at the beginning of the TNF domain, which is involved in ligand-receptor binding (S243X^(C-A)^); Figure 3G and Figure S3). An analysis of the location of *eda* in the zebrafish radiation hybrid map placed *eda* on LG5**.** Subsequent linkage analysis of the *Nkt* mutation and markers indicated by radiation hybrid analysis demonstrated tight linkage of the mutant to this region (Figure 3F); the S243X mutation was always found in fish with the *Nkt* phenotype and served as a consistent genotypic marker.

### Role of Ectodysplasin Signaling in Regulating Epithelial Signaling Centers: Scale Placode Formation

In fish, scales are bony elements that develop in the dermis underlying the epidermis. In amniotes, most integumentary organs affected by loss of Eda signaling have structural derivatives stemming from the epidermis (*e.g*. specific keratins of hair, feather and nail, secretory cells of glands). These integumentary organs develop from reciprocal signaling interactions between the basal epidermis and subjacent mesenchyme often controlled by a regional epithelial thickening called the epidermal placode. Eda signaling is necessary for the development and patterning of epithelial placodes of many integumentary organs in both the mouse and chick [19]–[22]. Expression of *Eda* and *Edar* is found predominantly in the basal epidermal cells, but in the case of feathers *Eda* is detected in the subjacent mesenchyme as well [21],[22]. Whereas expression of developmental signaling genes such as *sonic hedgehog (shh)* in the development of integumentary appendages are comparable between vertebrates [23], evidence for an early developmental role of the epidermis in induction or patterning of the teleost scale is lacking. The formation of an epithelial placode and signaling center in the development of amniote integumentary appendages is associated with histological changes in the basal cells of the epidermis; a similar structure has not been described in fish epidermis [24]. As early teleost scale development is quite different to that of other vertebrate integumentary organs, such as hairs and feathers, we addressed the question whether Eda signaling had a similar function in the epidermis of zebrafish during scale formation.

**Figure 4:**
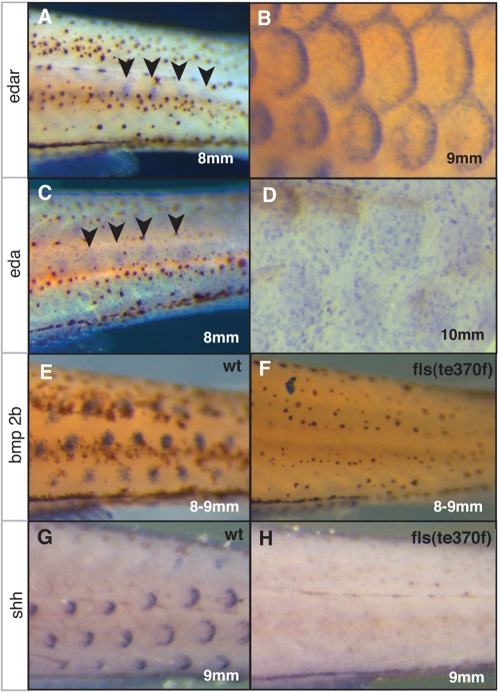
The role of *edar* in expression of developmental patterning genes during early scale development. Expression of *edar* (A, B) and *eda* (C, D) in early forming scales; arrowheads indicate site of expression of initial forming scales. A) *edar* expression above site of scale formation in 8 mm long (approximately 30 dpf juvenile fish) and in larger juveniles (9 mm; 30 dpf). C) *eda* expression during early scale development on the flank (8 mm) and in forming scales of older juvenile fish (10 mm, D). Expression of developmental genes *bmp2b* and *shh* in early scale development in wildtype (E, G) and *fls* ^te370f^ (F, H) juveniles (9 mm).

We detected the expression of both *edar* and *eda* in the skin of juvenile fish by whole mount *in situ* analysis (WMISH). The expression of both genes presaged the formation of the initial scale row along the flank just ventral from the midline mysoseptum in the caudal peduncle (arrowheads, Figure 4A and C; [24]). During scale formation, the expression of *edar* becomes progressively restricted to the posterior margin (Figure 4B) while *eda* expression persists throughout the scale primordia (Figure 4D). Developmental genes *shh* and *bone morphogenic protein 2b* (*bmp2b*), whose orthologues are known to be essential for placode development in the mouse and chick, show similar placodal expression as seen with *edar* (Figure 4E and G). Analysis of *shh* and *bmp2b* expression in *fls* ^te370f^ indicated the necessity of *edar* function for their expression (Figure 4F and H).

We investigated the development of scale primordia in wild type and mutant *fls* ^te370f^ fish by light and transmission electron microscopy. Previous detailed histological work found evidence for raised signaling activity in the epidermis as measured by increased endoplasmic reticulum (ER), and secretory activity of the basal epidermal cells prior to scale formation [23]. However these changes in the basal epidermal cells were not associated regionally with sites of scale formation nor was there any indication of histological changes in basal cell morphology that are associated with placode formation in other vertebrates. To our surprise, in wild type juvenile fish, we discovered the formation of histologically defined, localized assemblies of cells of the basal epidermis that resemble early stages of the formation of hair and feather placodes.

**Figure 5:**
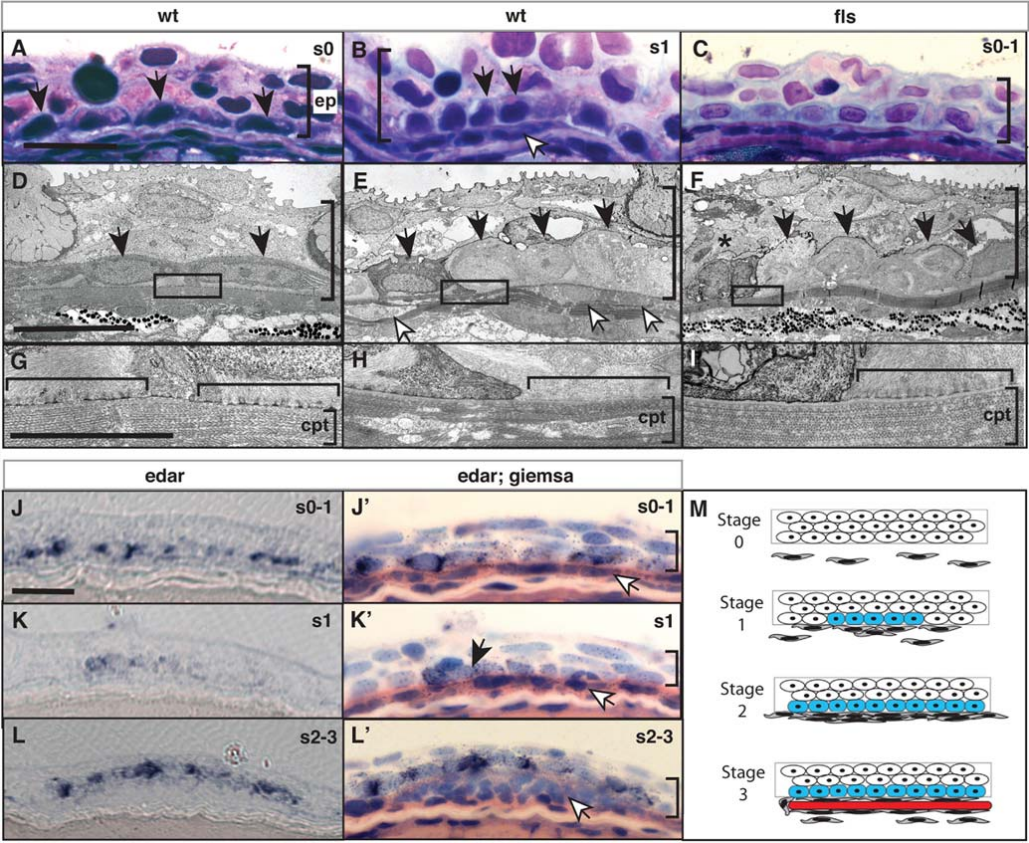
Eda signaling regulates the formation of an epidermal placode during scale development. Histological analysis of wild type (A, D, B, E) and *fls* ^te370f^ (C, F, I) integument of 8 mm standard length. In wild type juveniles (B, E), basal epidermal cells (black arrow heads) show a heightened, and cuboidal morphology at sites of scale development as indicated by an accumulation of migrating fibroblast-like cells (white arrowheads). (H) This morphology of the epidermis is associated with a reworking of the collagen layer of the stratum compactum (*cpt*; [24]). This is in contrast to the flattened morphology of basal epidermal cells lateral to those of the scale placode (A, D) and underlying dense stratum compactum (G). In *fls* ^te370f^ this basal epidermal structure is disorganized and cell morphology is disrupted (C, F) including evidence of cell death (asterisk). The lack of reworking of the collagen of the stratum compactum in the *fls* ^te370f^ mutant is associated with retention of hemidesmosomes (horizontal bracket G–I). *edar* is expressed in cells of the wildtype epidermis (J, K, L). Counterstaining of the same sections confirms the expression in basal cells overlying initial accumulating fibroblasts (white arrowheads; J′, K′, L′). Expression of *edar* is observed prior to organization of the placode and fibroblast aggregation and maintained in cells of the epidermal placode through early scale development (J–L). M) Schematic depicting scale development and *edar* expression. The stages of scale development are modeled using analogous stages as described for hair development [35]; stage 0, nascent epidermis; stage 1, placode specification; stage 2, scale pocket; stage 3, matrix deposition and ossification. Blue, *edar* expression; red, scale formation. *ep*, epidermis; *cpt* stratum compactum. The vertical bracket demarcates the extent of the epidermis in the sections. Measurement bar equals 10 μm.

Prior to the development of the scale, the dermis consists of compact collagen layers (stratum compactum) and scattered dermal fibroblasts [24]. The epidermal basal cells have a uniform elongate morphology (black arrows, Figure 5A, D) with high levels of basally located intermediate fibrils (Figure 5G). At the initiation of scale development, there is an accumulation of fibroblasts subjacent to the basal epidermis, associated with a reworking of the collagen strata [24]. We find a specific alteration in the morphology of the basal epidermal cells in wild type juvenile fish that coincides with the initial accumulation of fibroblasts at the sites of scale development (Figure 5B, E). These basal cells become cuboidal and have decreased width, such that they form a unit of closely packed cells (black arrows, Figure 5B, E). This is observed above the localized accumulation of fibroblasts in the dermis (white arrows, Figure 5B, E). In addition, in these placodal-like cells, the ER appears less prominent (data not shown), and hemidesmosomes, the cellular junctions involved in the attachment of the basal epidermal cells to the basal lamina, are almost completely absent (brackets, Figure 5H and I). In contrast, the adjacent lateral epidermal cells show high levels of both ER and hemidesmosomes (data not shown and Figure 5G brackets, respectively).

In *fls* ^te370f^ juvenile fish, at a corresponding site on the flank as in wild type, we detected the formation of similar aggregations of basal epidermal cells (black arrows. Figure 5C, F). However, unlike the structures found in the wild type zebrafish, the epidermal cells of the placode were disorganized and showed histological evidence of cell death (Figure 5F). As is the case in wild type, the epidermal basal cells in the placode of *fls* display a reduced ER, however hemidesmosomes are present in the same high numbers as in adjacent cells in wildtype (brackets, Figure 5H compared to brackets Fig. 5I). Lateral basal epidermal cells in *fls* ^te370f^ */edar* showed elongate morphology similar to those of their wild type siblings (data not shown). The expression of *edar* is seen in the basal cells of forming scale placodes (Figure 5J–L; stages 1–3 Figure 5M) arising during early specification of the scale placode (s1; arrowhead Figure 5K). We were unable to detect *eda* expression in sections due to the weak hybridization signal.

These data support the notion that an epidermal placode is involved in dermal scale formation. Further we find that the epithelial organization and function of the developing scale placode is dependent on *edar*.

### Role of Ectodysplasin Signaling in Regulating Epithelial Signaling Centers:Maintenance of the Fin Fold and Establishing Anterior-Posterior Polarity of the Developing Fin

**Figure 6:**
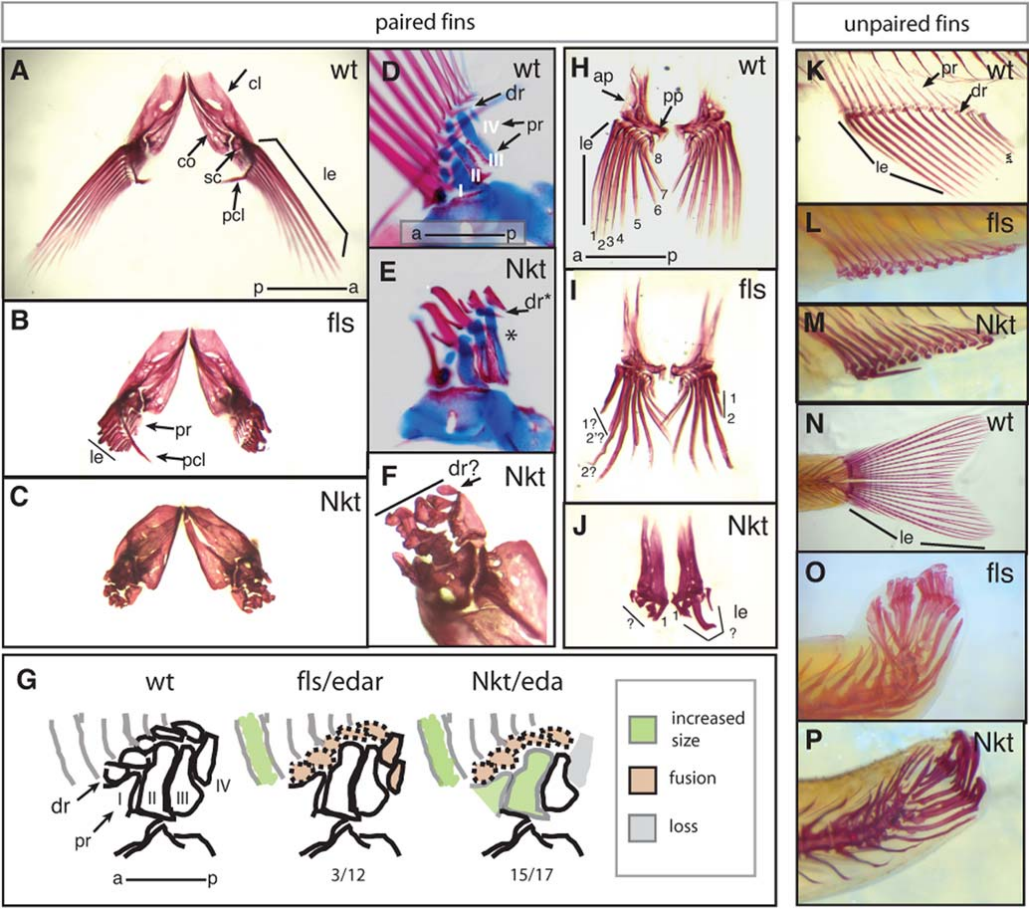
Fin development is defective in *fls* and *Nkt* mutant zebrafish. Alizarin red stained adult zebrafish fins show a drastic effect of *fls* ^te370f^ and *Nkt* on development of the lepidotrichial dermal rays of both the paired and unpaired fins. A–C,F) Pectoral fins, anterior-dorsal view; D, E) double staining developing pectoral fins with alcian blue and alizarin red show early patterning of the endochondrial bones of the pectoral fin of size matched wild type and *Nkt* homozygotes (asterisk indicates loss of fourth proximal radial). G) effect of *fls* and *Nkt* mutants on the patterning of the pectoral fin skeleton scored as the number of specimens showing alteration in pattern or form over total analyzed. The identity of the proximal radials is noted (I–IV). H–J) Analysis of pelvic fin development in *fls* and *Nkt* mutants. Numbers denote anterior-posterior identity of the lepidotrichia. K–M) Defects in the formation of the lepidotrichia in adult anal and (N–P) caudal fin of *fls* (L and O) and *Nkt* (M and P). *ap*, ascending process; *cl*, cleithrum; *co*, corticoid; *dr*, distal radial; *sc*, scalpula, *le*, lepidotrichia; *pcl*, postcleithrum; *pr*, proximal radial.

The phenotype of both *Nkt* and *fls* demonstrate that Eda signaling is necessary for fin development. The growth and patterning of lepidotrichia are affected in all fins. Lepidotrichia are specified, however fail to maintain growth and elaboration of the fin rays (Figure 6A–C, H–P). Unpaired fins showed no defects in patterning of the endochondrial bones of the proximal and distal radials (Figure 6K–P); the dorsal pitch of the caudal fin is an indirect effect of the mutation on swimming without fin rays (amputated fins that fail to regenerate show similar morphology). In *fls* adults, fusions of the distal radials of the pectoral fin are seen at a low penetrance (Figure 6G, data not shown). In *Nkt*, there is an increase in the frequency of patterning and growth defects of the endochondrial components of the fin (Figure 6G). These alterations include the loss of the fourth proximal radial, altered growth patterns of anterior proximal radials 1 and 2, as well as lack of articulation of the distal radials (Figure 6 D–F). *Nkt* causes a strong effect on lepidotrichial growth of both the pectoral and pelvic fins (Figure 6E–F, J). In contrast, a specific effect on the growth of anterior lepidotrichia of the pelvic fin is seen in *fls* where the dermal rays of the anterior (*e.g.* 1,2) are significantly shorter than rays at equivalent positions in wild type (Figure 6I). The asymmetry of lepidotrichial development suggests that, like the proximal endochondrial fin skeleton, the fin rays have a specific regional identity to provide the shape and form of the fin.

Early limb development is driven by a localized organization of epithelial cells at the distal tip of the forming limb, termed the apical ectodermal ridge (AER) [25]. In zebrafish, the AER is involved in larval patterning of the paired fins, while the later stages of fin development are organized by an analogous epidermal formation of the fin fold in both paired and unpaired fins [26],[27]. In tetrapod limb development anterior-posterior specification is controlled by posterior mesenchyme expressing *Shh*. The function of this zone of polarizing activity (ZPA) expressing *Shh* is maintained by reciprocal signaling interactions between the ZPA and the AER. This interaction is necessary for proper patterning and growth of the tetrapod limb. In the zebrafish, *shh* and signals from the AER also orchestrate patterning and outgrowth of the early fin buds [28]–[30]. In addition, genes functioning early in fin development, such as *shh*, play important roles during late fin development regulating growth and branching of lepidotrichia growth [31].

**Figure 7:**
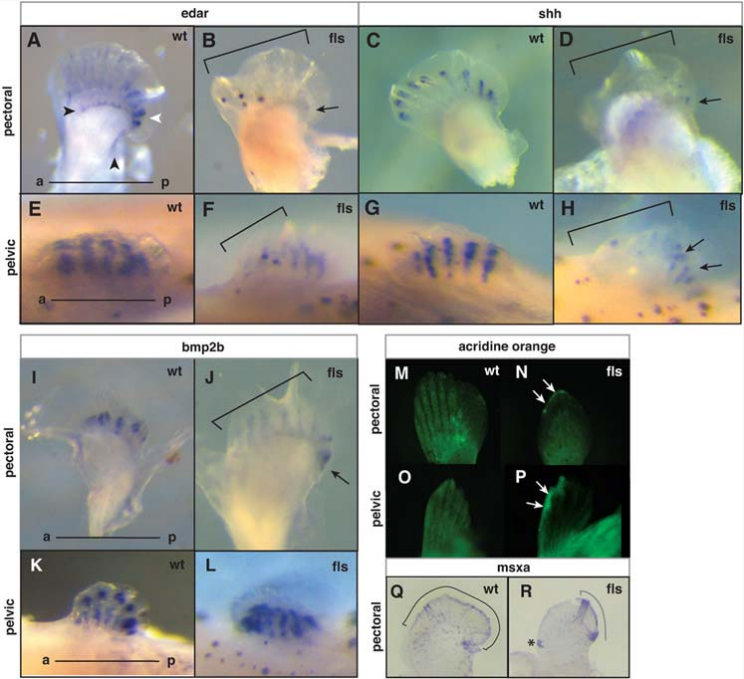
Eda signaling and the maintenance of anterior-posterior pattern in late paired fin development. Analysis of *edar* (A–B, E–F), *shh* (C–D, G–H), and *bmp2b* (I–L) expression in developing pectoral (A–D, –J) and pelvic fins (E–H, K–L) from 8 mm juvenile fish of wild type (A, E; C, G; I, K) and *fls* ^te370f^ mutant fish (B, F; D, H; J, L). A–B) Arrowheads indicate two distinct patterns of *edar* expression in the pectoral fin: an expression that marks the posterior edge and distal region of the development of the proximal radials (black); and a posterior bias of *edar* expression in the forming lepidotrichia (white). Arrows point out the remaining posterior expression in mutant fins. Brackets in all panels outline anterior deficiencies in gene expression in *fls* mutant fins. M–P, analysis of patterns of cell death in the developing paired fins by retention of acridine orange stain. N, P) Arrows point out anterior distal regions of cell death in both pectoral and pelvic fins from the mutant; (M, N) pectoral fin and (O, P) pelvic fin respectively. Q, R) Expression of *msxa* in wild type and mutant pectoral fins. Region of expression outlined with brackets; asterisk marks an ectopic site of expression.

We investigated the regulation of *edar* in mid to late fin development focusing on the development of the paired fins. In early fin fold stage of pectoral fin development (8 mm), we detected *edar* expression in both the distal margin of the endochondrial radials (black arrowhead, Figure 7A) as well as more distally in the forming lepidotrichial rays (Figure 7A). The expression of *edar* in the fin fold had a posterior bias in wild type fins (Figure 7A, white arrow). The pelvic fin showed similar expression of *edar* in forming lepidotrichial rays (Figure 7E). *shh* and *bmp2b* expression was observed in the forming lepidotrichia of both the pectoral and pelvic fins of wild type juveniles, having a similar distal bias in the leading margin of all rays (Figure 7C, G and I, K, respectively). Expression of all three genes in *fls* was decreased in the anterior portion of the pectoral fins (Figure. 7B, D, J). However, residual expression of all three genes was found in the posterior margin of the fin (Figure 7B, D, J arrow). In the pelvic fins, similar loss of anterior expression of *edar* (Figure 7F) and *shh* (Figure 7H) was seen in the *fls* mutant. We did not detect any difference in *bmp2b* expression in the pelvic fins even though obvious morphological differences in the developing rays of the samples could be seen (Figure 7L).

We asked if the alteration of polarity of gene expression in the *fls* mutant was associated with regional cell death. Using acridine orange uptake as an assay for cell death (*e.g.* [32]), we analyzed fins of *fls* and siblings at size matched stages (7–9 mm) for regional patterns of cell death. In 7 mm juveniles, we detect a differential retention of acridine orange between *fls* and siblings in the anterior, and anterior-distal margin of the developing pectoral fin (Figure 7M–N). Similarly, in the pelvic fin of 8–9 mm juveniles, retention of the label was seen in the anterior distal margin of the fin (Figure 7O–P). Consistent with these data suggesting asymmetrical loss of the fin fold epidermis, we find that *msxa*, a marker of the distal epithelium, is differentially expressed in the mutant (Figure 7Q and R).

**Figure 8:**
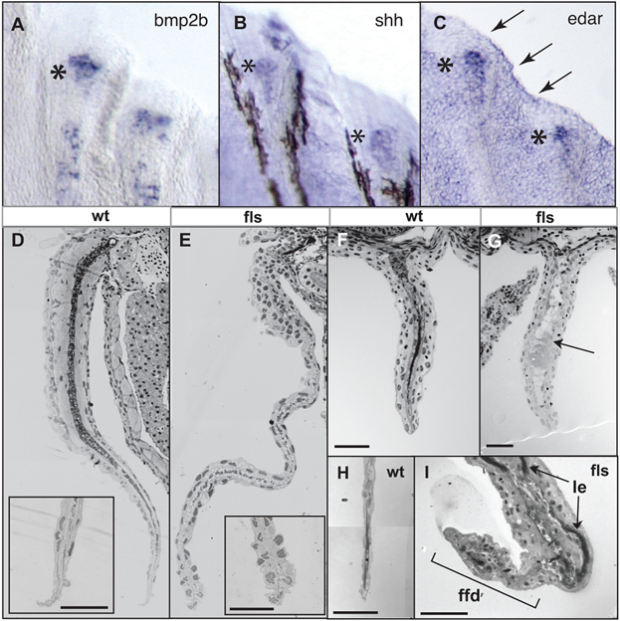
Eda signaling is required for the function of the fin fold during late fin development. Expression of *bmp2b* (A), *shh* (B) and *edar* (C) transcripts in developing juvenile (8 mm, 30 dpf) fin rays of the caudal fin; asterisks indicate regional expression within distal tip of developing ray; arrows in (C), expression in distal epidermis of the fin fold. D–I) Histological analysis of both paired (pectoral, D, E) and unpaired fins (anal F, G; and caudal, H, I) from *fls* ^te370f^ and wild type siblings. *fls* ^te370f^ fins showed a general deficiency in the maturation of the muscle and dermis of the fin (arrow G; acellular debris in anal fin of *fls*). Insets (D, E), tip of fin at higher magnification showing degeneration of the nuclei of the epidermis in the mutant fin. *ffd*, fin fold; *le*, lepidotrichia of the fin rays.

We next analyzed gene expression during late development of the lepidotrichia. The expression of *edar* during late fin development was observed in forming lepidotrichia of all fins with a distal bias in its expression (Figure 8C). The expression in the forming dermal ray was similar in both location and timing to that of *bmp2b* and *shh* (Figure 8A–B, asterisk). In addition, *edar* was found expressed proximally between forming rays and at the distal margin (Figure 8C arrow). We were unable to resolve a clear signal for *eda* in the forming fins using WMISH. The expression of *edar* in the distal lepidotrichial tips suggests a late developmental role of Eda signaling in regulating formation of the lepidotrichia in concert with *shh* and *bmp2b*.

Histological analysis of *fls* mutant fins at an early stage of lepidotrichial formation reveal a general deficiency of the development of the entire mesodermal component of the fin such as cartilage and muscle in both the paired (pectoral fin, Figure 8D, E) and unpaired fins (anal and caudal fins, Figure 8F–G; H–I, respectively). In contrast, the epidermis of the fin is formed and is similar to that of size matched siblings. However, close inspection of the distal tip of the fins showed disorganization of the epidermis and degeneration of distal epidermal nuclei (insets Figure 8F, J). From these analyses, we hypothesize that loss of *edar*-mediated signaling leads to a defect in mesenchymal cell proliferation, muscle cell migration and defective lepidotrichial growth in the fin that correlates with degenerative defects seen in the distal epidermal fin fold.

## Discussion

We used a forward mutagenesis approach in the zebrafish to investigate the developmental mechanisms that underlie changes in adult form. Here, we identified a role of Eda signaling in the development of the dermal skeleton in the adult zebrafish. Mutations in either *Eda* or *Edar* have been shown previously to cause defects in integumentary appendages in several mammalian species. Additionally, Eda signaling genes have been associated with variation in morphology that occurred during the evolution of teleost fishes *(eda)* and in variation of human populations *(Edar)* [33],[34]; see below). Thus, through a forward genetic approach, we were successful in identifying genes that are important for the development and variation in adult form. We further show that the ENU generated alleles of *fls*, *t3R357W* and *dt3Tpl*, affect similar residues as those mutated in familial cases of HED [11],[12],[18] supporting the utility of adult zebrafish mutants as models for the investigation of heritable human disease.

### Conserved and Ancestral Role of Eda Signaling in Vertebrate Development

We show that Eda signaling is necessary for the development and patterning of the dermal bones of the skull, scales, fin rays as well as teeth of the adult zebrafish. The correlated effect in these zebrafish structures is due to a developmental role of Eda signaling in organizing epithelial cells into signaling centers. In the case of scale development, Eda signaling is necessary for the basal epidermal cells to form a functional placode. Epidermal placodes are involved in the formation of integumentary appendages of other vertebrates such as hair, glands, feathers and teeth. These structures have been shown to act as signaling centers to orchestrate appendage development. We speculate that a primary function of Eda signaling in scale development is to promote cell-cell adhesion within the placode and that the coordinated signaling of the placode induces fibroblast assembly in the underlying dermis, an early step in scale formation.

Schmidt-Ullrich et al. documents the formation of the hair placode and outline a stage series of placode formation in the mouse [35]. They report that the *downless* mouse mutant, which has a mutation that disrupts the mouse *Edar* gene [36], causes arrest of placode formation at a pre-placode stage of development (P0–P1). This stage closely resembles the stage of scale placode formation that is affected in *fls* shown here. In agreement with our findings, Schmidt-Ullrich et al. further note a reduction of cell-to-cell adhesion within the placode and find increased apoptosis in the absence of Edar function. This suggests that there is a conserved developmental role of Edar between dermal scales of fish and mammalian hairs. During normal hair development, the hair placode invaginates to form the hair bulb. By contrast, the post-placodal events of scale formation in fish do not involve morphogenetic changes of the epidermis, rather the accumulation of mesenchymal cells subjacent to the epidermal placodal cells to form the scale pocket. Thus, Eda signaling in mammals and teleosts is conserved in the early phases of placode formation in controlling the functional continuity and signaling of the epidermal placode to orchestrate appendage formation. However, the downstream interpretation of the epithelial-mesenchymal signaling differs beyond this point leading to altered morphogenetic responses and histological differentiation to form diverse appendages such as scales and hair.

In the fin, Eda signaling directs late stages of fin development such as the formation and growth of the dermal rays. The effect of loss of *edar* function on fin development uncovers an intrinsic developmental polarity of the late developing fish fin. This is seen both in the development of the proximal endochondrial bones as well as in the formation of the fin rays. We find that the change in patterning in the mutants is correlated with asymmetrical cell death of the distal marginal fin fold as well as a reduction of *shh* expression. This finding is similar to the effect of loss of AER function resulting in anterior-distal cell death and reduction of Shh activity in tetrapod limbs [37]–[39]. While there has not been any previous indication of a role of Eda signaling in tetrapod limb development, both the expression of *Edar* and related receptor, *Troy*, have been detected in the AER of mice [40],[41].

A second developmental role of Eda signaling in the developing fin is observed in the outgrowth and patterning of the individual lepidotrichial rays evidenced by expression of *edar* in the distal tip of the forming rays and distal epidermis. The expression of *edar* is again associated with that of *shh* and *bmp2b*. The expression of *shh* and *bmp2b* has been shown to be within the basal epidermis overlying the forming lepidotrichia [31]. Given the expression of *edar* during fin development and the defects observed in the distal epidermis in the mutant, it is likely that the function of Eda signaling is to maintain the growth permissive function of the fin fold through its regulation of a distal signaling center of individual rays. The concomitant expression of *edar*, *shh* and *bmp2b* in both distal lepidotrichia development and during placode specification suggest that they work in concert to mediate the inductive and/or permissive effects of the epidermis – thus organizing signaling centers for the development of the dermal skeleton.

While the nature of the defect in tooth formation or dermal bone patterning of the skull in the *fls* and *Nkt* mutants has not been characterized in detail, there is evidence that inductive signaling from the pharyngeal epithelium or cranial epidermis is necessary for appropriate development of both tooth [42] and skull [6], respectively; Eda signaling likely shares a common role in inductive signaling in each of these diverse organs.

### Genetic Commonalities of Eda Signaling: From Fish to Man

Mutations in the *EDAR* and *EDA* genes underlie a large percentage of autosomal and X-linked HED in humans, respectively [18],[43]. In the case of *EDAR*, both recessive and dominant mutations are associated with the HED phenotype in humans, however dominant mutations are found only within the death domain of the protein. These mutations are believed to act in a dominant negative fashion, although by unknown mechanisms [44]. We see similar dominance of a *fls* allele that affects the death domain of *edar* while all *fls* mutations outside this region do not show a dominant phenotype. Autosomal dominant HED in humans caused by mutation of *EDAR* within the death domain displays a large degree of phenotypic variability [12]. For example, the I418T mutation in human, which affects the same amino acid as *fls* ^dt3Tpl^ (I327F), shows distinct phenotypic variability depending on genetic background [18]. Interestingly, the *fls* ^dt3Tpl^ zebrafish mutant displays similar dominance and variation as the human allele affecting the same residue. These findings suggest that the molecular mechanisms of Edar function are similar between fish and humans.

X-linked HED caused by mutations in the human *EDA* gene represents the majority of cases of this disease [43],[45]. The zebrafish *Nkt* mutation described here is affected in the TNF domain and shows a mild dominant phenotype (S243X). As the *EDA* gene is sex linked in humans the molecular nature of different alleles can not be analyzed since the allele will be hemizygous in males and mosaic in female carriers. The zebrafish *eda* gene is autosomal in the zebrafish. Thus, *Nkt* exposes previously unknown dominant function of mutations in this gene since a true heterozygous condition is formed. Hemizygous wildtype condition in humans indicates that the dominance we see in *Eda* is probably not due to haploinsufficiency. Since EDA functions as a homotrimeric protein [46], a plausible mechanistic explanation for the observed dominance of *Nkt* is that the C-terminal truncation inhibits the function of the wild type protein in binding to Edar.

### Eda Signaling and the Development and Variation of Adult Form

Mutations affecting Eda signaling lead to impaired development of integumentary appendages of fish, birds, and man. These changes lead to viable changes in adult morphology. Mutations disrupting Eda signaling have been described for another teleost species. The spontaneous *rs-3* mutant in medaka (*Oryzias latipes*), is shown to have a transposon insertion in the 5′ UTR of *edar* resulting in the reduction of scales but no effect on fin or teeth development [47]. The zebrafish mutations described here show a previously undescribed role of Eda signaling in the development of the fins, teeth, as well as dermal bones of the skull – phenotypic traits observed in human alleles but not reported in the medaka mutant. As the phenotype of the *rs-3* mutant is similar to the *fls* ^dfang^ allele in the zebrafish, it is likely that the more subtle phenotypes observed in the medaka mutant is due to partial loss of function of *edar* caused by a hypomorphic *rs-3* allele [47].

The graded effects seen in the expressivity of mutations affecting *eda* and *edar* points to a dose sensitive readout of the Eda signaling pathway that affects different organ systems with varied expressivity. In the dominant *fls* ^dt3Tpl^ or *Nkt* heterozygotes, the shape and number of scales and teeth as well as patterning of the skull are affected, however there is no change in fin ray development. Similarly, the *fang* allele of *fls* clearly demonstrates this dose sensitivity as functional copies of Edar are titrated by the concomitant use of a new splice site in the mutant leading to the reduction in the amount of wild type transcript made (Figure 3D). This reduction in the amount of *edar* transcripts cause defects in scale and tooth development, however fins are normal. *fang/te370f*, in which the *fang* allele is in *trans* to a presumed null, further reduces the relative levels of wild type *edar* transcripts leading to further reduction of both teeth as well as fin lepidotrichia. Similar dose sensitive responses to levels of EDA signaling are seen in tooth development of the mouse regulating the number and shape of teeth [48],[49]. There are several reports of hypodontia in humans resulting from altered EDA function that do not show other phenotypes such as hypothrichosis or nail defects [50]–[52]. Given our findings, it is likely that these particular alleles are hypomorphic and this is sufficient to explain the differential organ sensitivity to levels of EDA signaling during development. These data indicate that control of the level of Eda signaling in post-embryonic development is an essential component for the determination of the number and form of many different organ systems of the adult.

Supporting this finding, we observed significant modification of expressivity of *fls* ^dt3Tpl^ in different genetic backgrounds indicating the existence of genetic modifiers of Eda signaling. This sensitivity of Eda signaling to genetic modifiers occurs in other teleost fish as well. In our analysis of the medaka *rs-3/edar* mutant, we find a high degree of variability in the extent of scale formation (Figure S4) suggesting the existence of background modifiers of Edar function in this species. Additionally, evidence from the stickleback, *Gasterosteus aculeatus*, suggests that genetic variance at the *eda* locus underlies differences in the extent of dermal plate formation in diverged populations of this species [33]. A quantitative trait analysis (QTL) of lateral plate formation in a low-plated form of the stickleback indicates a significant modification of the reduced plate phenotype (*eda* locus) with modifying effects within and between loci affecting plate number and size [53],[54]. Interestingly, recent evidence also shows a significant association between the *edar* locus and dermal plate number in sticklebacks in addition to the predominant *eda* locus [55]. Thus variation at these gene loci may act in concert to regulate number of dermal plates/scales.

Thus, while loss of Eda signaling can lead to severe phenotypes, the phenotypic consequences of variation in Eda signaling are graded and canalization of Eda signaling is prevalent. Therefore, buffering of the phenotypic outcome that results from defective Eda signaling could be a common mechanism that permits viable and diverse phenotypes. These viable phenotypic variations then could serve as a basis for selection. The lack of a coding change at the *eda* locus in sticklebacks that is associated with the loss of dermal plates has lead to the argument that, in this case, evolution of this trait is due to changes at *cis*-regulatory elements controlling *eda* expression [33]. Our findings on the dose and organ specific sensitivity of Eda signaling in different structures of the zebrafish argues that evolution of this trait could result from a regulation of absolute levels of expression.

Interestingly, recent analysis of single nucleotide polymorphism (SNP) frequency in human populations supports the role of Eda signaling in causing phenotypic variation. Analysis of SNP variation between diverse human populations shows evidence of selection of the *EDAR* locus in East Asian and American populations [56],[57]. A defined allelic variant of *EDAR* within these populations leads to a coding change in the death domain of EDAR and is a candidate allele for altered gene function that could have lead to the region being fixed in these populations [34]. There is evidence from association data that this allele is associated with thick hair in these populations [58], however the full extent of phenotypes that are affected in these populations that are related to EDA signaling has not been analyzed. It is interesting to note that recent work has identified this allele of *EDAR* as having an enhanced effect on Eda signaling in mouse models containing the altered human residue [59]. Given that variation in the number and shape of integumentary derivatives of the dermal skeleton are a common morphological change in teleost evolution *e.g.* [60], it will be important to further investigate the prevalence and type of genetic changes in Eda signaling genes in cases of natural variation of these adult characters.

### Mutagenesis and Allele Designation

Zebrafish mutagenesis was performed following [61] with 5 treatments of 3.3–3.5 mM ethylnitrosourea. Screen design was similar to that described [62]. Allele designation was determined using standard nomenclature with the addition of the molecular lesion or phenotypic description (when appropriate) to the designation. The serial numbers of the mutants found in the ZF models screen are as follows: *fls* ^t3R367W^ (#0621); *fls* ^dt3Tpl^ (#1248); *Nkt* ^dt3S243X^ (#1261). Information on the screen can be found at http://www.zf-models.org/. The screen for additional *fls* alleles used mutagenized *TLF* founder males treated similarly as Tü males used in the screen.

### Mapping

Rough mapping of F2 progeny against a reference panel of SSLP markers [63] indicated that *fls* was located on linkage group (chromosome) 9 (LG9) with loose linkage to z20031 (61.3cR; Figure 3A). We found *fls* to be closely linked to markers z7001 and z11672. Results from radiation hybrid screening indicated linkage of zebrafish *edar* to markers positioned on LG9 in the region predicted by initial mapping analysis. Analysis of flanking markers and internal polymorphisms in *edar* showed tight linkage of the *fls* mutation to the *edar* gene. Using the defined molecular differences between WIK and Tü strains, we did not find recombination in 238 meioses indicating that the mutation was located less than 0.4 cM away.

### Cloning and Sequence Analysis

We isolated the full-length cDNA of zebrafish *edar* and *eda* by reverse transcription (RT) PCR using sequences provided from genomic alignments and subsequent amplification of the cDNA ends by rapid amplification of DNA ends (RACE). cDNA was generated from RNA from blastemas of amputated caudal fins that had been allowed to regenerate for two days. cDNA sequences of zebrafish *edar* and *eda* genbank accession numbers are EF137867 and EF137866, respectively. Protein alignment of Edar and Eda were generated by ClustalW alignment (http://www.ebi.ac.uk/clustalw/) and Box Shade software (http://www.ch.embnet.org/software/BOX_form.html) using a 0.4 identity threshold. *Edar* and *eda* sequences of other species were obtained from genomic databases at NCBI (http://www.ncbi.nlm.nih.gov/), Sanger (http://www.ensembl.org/index.html), and Tigr (http://www.tigr.org/tdb/tgi/).

Real Time PCR was performed on cDNA obtained from blastemas from two day old caudal fin regenerates. Calculations were made from three biological replicates and three technical replicates according to [64]. Primers were designed for wild type specific transcripts by using sequence from the neighbouring exon borders that are not adjacent to each other in Fang-transcripts. Fang specific primers were designed against the fang specific transcript sequence, which is spliced out in wild type. Crossing points of the control reaction, which were higher than in the water control, were set to the value for water. Normalization was done against the efficiency of primers to β-actin.

### Bone Stain and Measurements

Adult bones were stained with alizarin red. Embryos were fixed in formalin (3.7% formaldehyde), briefly dehydrated in 70% ethanol, and placed in 1 g/l alizarin red; 0.5% KOH until bones suitably stained. Fish were destained in 1% KOH until background stain was lost and subsequently cleared in glycerol for analysis. For analysis of forming cartilage, fish were prestained in alcian blue from 4–24 hours. The fish were then destained, lightly trypsinized (3 g/l; 37°C) and processed for alzarin red staining.

Skeletal measurements were made using digitizing software from Zeiss using a dissecting microscope. Measurements were made from fixed landmarks on each axis of the skull that did not vary depending on position of the suspensorium: the premaxilla was used for the distal most point on the length (L) axis, while the quadrate-anguloarticular joint was used as a ventral landmark for the height (H) axis. Raw measurement values are represented as normalized ratios of the distance along each axis in relation to the position of the center of the eye; values are normalized for standard length of the fish.

### Whole Mount In Situ Hybridisation and Immunostaining

Probes for whole mount *in situ* hybridisation were generated by reverse transcription from cDNA made from regenerating caudal fin tissue. Digoxigenin labeled RNA probes were purified using P-30 micro bio-spin columns before use (BioRad). WMISH protocol was performed as described [65] , at 70°C and with the addition of 0.1% CHAPS to hybridization and post hybridization wash buffers. Reactions were stopped in PBS, post-fixed and placed in methanol overnight to reduce non-specific staining.

Acridine orange (Sigma) was used as a marker of apoptosis in developing tissue [32]. Juveniles were immersed in fish water containing 5 μg/ml acridine orange for 5 minutes and then washed with fish water, anesthetized and post-fixed in formalin to assist visualization of staining.

### Statistical Methods

Analysis of cranial measurements were performed using Hotelling's T squared test for two dependent variables. For scale counts and size dimensions, a *t*-statistic for differential means was used to assess significance. Calculations and probability assessment were calculated using Biosoft 200 software (www.biosoft.com) and Excel statistical package.

### Electron Microscopy

Specimens of 8–9 mm juvenile fish were fixed with a mixture of 4% formaldehyde in PBS and 1–2.5% glutaraldehyde at room temperature and subsequently placed at 4°C. After post-fixation with 1% osmium tetroxide in 100 mM PBS for 1 h on ice, samples were washed with H_2_O, treated with 1% aqueous uranyl acetate for 1 h at 4°C, dehydrated through a graded series of ethanol and embedded in Epon. Ultrathin sections were stained with uranyl acetate and lead citrate and viewed in a Philips CM10 electron microscope. In addition, toluidine blue stained Epon sections of 0.5 or 3 μm thickness were prepared for light microscopy.

## Supporting Information

Figure S1 *fls* and *Nkt* alter size and proportion of the adult zebrafish skull. In addition to variations in integumentary structures, *fls* and *Nkt* exhibited a distinct change of the shape and size of the adult skull. Measurements of the absolute proportions of the adult skull, normalized for overall growth of the fish as determined by standard length, demonstrate that both *fls* and *Nkt* homozygous mutations result in overall larger skulls of the fish (*fls* ^te370f/te370f^, n = 16, T2 = 23.7, p<0.001; *Nkt*, n = 10, T2 = 64.1, p<0.001; *fls* ^te370f/dt3Tpl^, n = 6, T2 = 22.8, p<0.005). The dominant effect of *Nkt* and *fls* ^dt3Tpl^ seen in development of the scale pattern was not observed in the formation of skull size. However, an analysis of changes in the proportional development of the skull by measurements of the relative positioning of the eye within the skull (L1/L2, H1/H2; Panel A) showed a significant and dominant effect of *Nkt*, *fls* ^dt3Tpl^ on the patterning of the skull (Panel C). This effect was seen in *fls* ^te370f^ homozygotes as well and was not specific to particular alleles of *fls*. The alteration in skull size and shape in the mutants does not involve loss of a particular organ structure or specific bone, rather a change in proportions of the developing skull.
(3.39 MB TIF)

Figure S2 A comparison of *edar* sequence in representative vertebrates. Edar alleles *fls* ^dt3Tpl^ and *fls* ^t3R367W^ positioned above sequence. Sites of splicing defects of *fls* ^te370f^ and *fls* ^t0sp213^ alleles demarcated with ˆ marker. Yellow, TNFR domain; Grey, transmembrane domain; Green, death domain; Red, polymorphic sites in WIK mapping strain.
(0.04 MB DOC)

Figure S3 A comparison of *eda* sequence of representative vertebrates. Blue, transmembrane domain; Green, furin cleavage site; Yellow, TNF domain; asterisk *Nkt* ^dtS238X^ allele; |, deleted residues in alternate spliced form of Eda-2.
(0.04 MB DOC)

Figure S4 Scale formation and variation in the rs3/*edar* medaka mutant on the cs-2 background. (A) Alizarin-red stained rs3 medaka showed substantial scale formation and variation of the extent of scalation. (B) Wild type cs-2 strain scalation pattern.
(5.72 MB TIF)
