# Supplementary figures and images for: Zebrafish eda and edar Mutants Reveal Conserved and Ancestral Roles of Ectodysplasin Signaling in Vertebrates
Source: PLoS Genet. 2008 Oct 3;4(10):e1000206. doi: 10.1371/journal.pgen.1000206 (PMC2542418; doi:10.1371/journal.pgen.1000206)

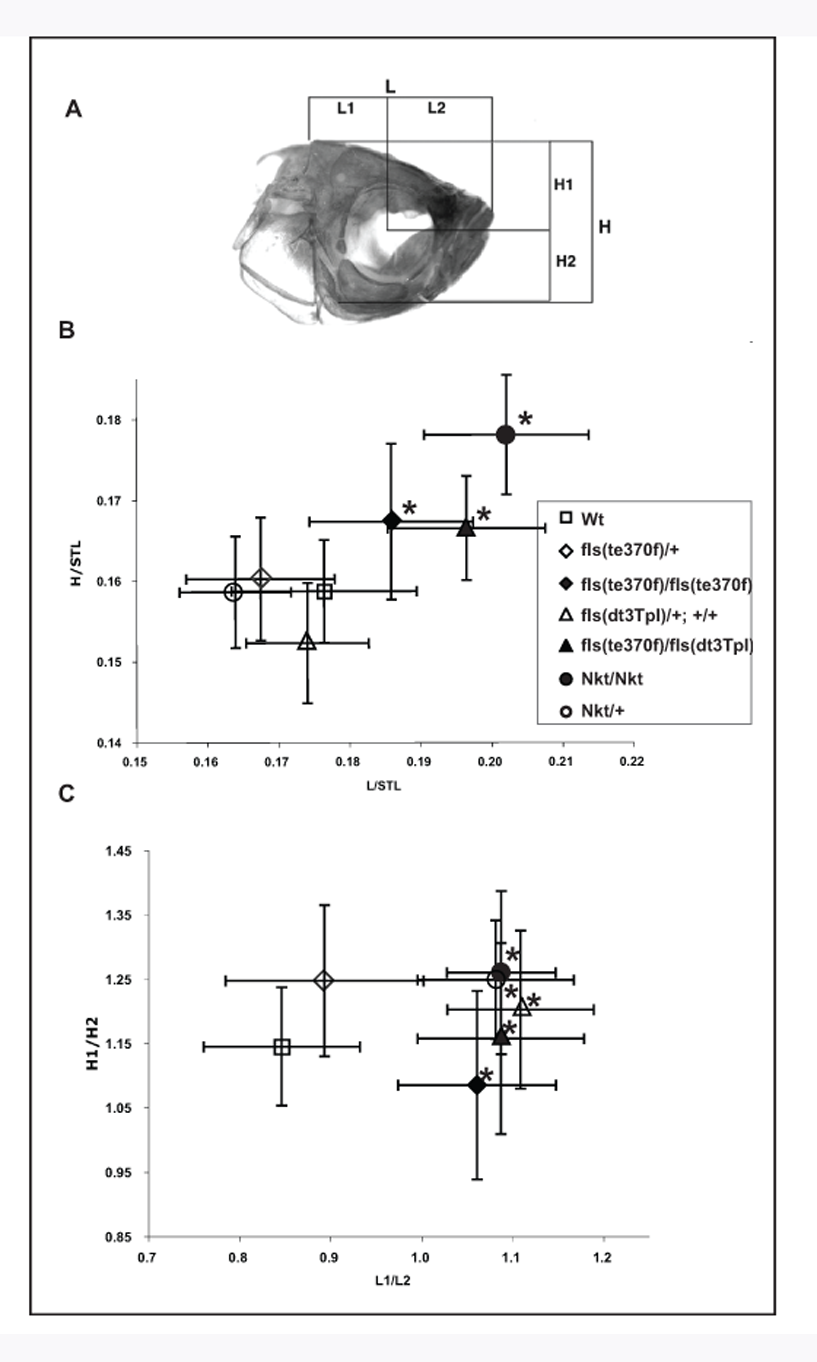

Supplement: Figure S1 — fls and Nkt alter size and proportion of the adult zebrafish skull. In addition to variations in integumentary structures, fls and Nkt exhibited a distinct change of the shape and size of the adult skull. Measurements of the absolute proportions of the adult skull, normalized for overall growth of the fish as determined by standard length, demonstrate that both fls and Nkt homozygous mutations result in overall larger skulls of the fish (fls te370f/te370f, n = 16, T2 = 23.7, p<0.001; Nkt, n = 10, T2 = 64.1, p<0.001; fls te370f/dt3Tpl, n = 6, T2 = 22.8, p<0.005). The dominant effect of Nkt and fls dt3Tpl seen in development of the scale pattern was not observed in the formation of skull size. However, an analysis of changes in the proportional development of the skull by measurements of the relative positioning of the eye within the skull (L1/L2, H1/H2; Panel A) showed a significant and dominant effect of Nkt, fls dt3Tpl on the patterning of the skull (Panel C). This effect was seen in fls te370f homozygotes as well and was not specific to particular alleles of fls. The alteration in skull size and shape in the mutants does not involve loss of a particular organ structure or specific bone, rather a change in proportions of the developing skull. (3.39 MB TIF) [file pgen.1000206.s001.tif]

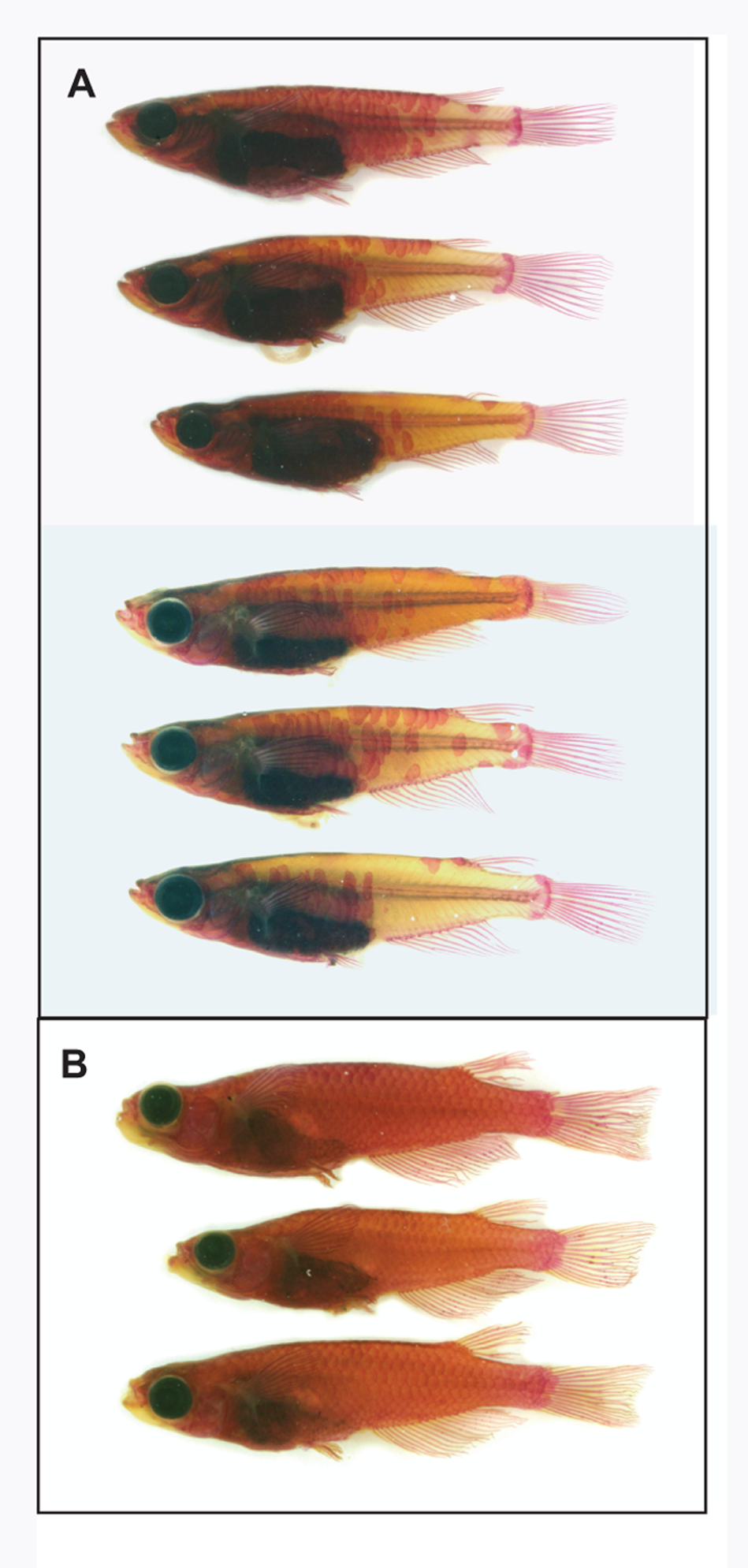

Supplement: Figure S4 — Scale formation and variation in the rs3/edar medaka mutant on the cs-2 background. (A) Alizarin-red stained rs3 medaka showed substantial scale formation and variation of the extent of scalation. (B) Wild type cs-2 strain scalation pattern. (5.72 MB TIF) [file pgen.1000206.s004.tif]
