# Supplementary material for: Zebrafish eda and edar Mutants Reveal Conserved and Ancestral Roles of Ectodysplasin Signaling in Vertebrates
Source: PLoS Genet. 2008 Oct 3;4(10):e1000206. doi: 10.1371/journal.pgen.1000206 (PMC2542418; doi:10.1371/journal.pgen.1000206)
Supplement: Figure S2 — A comparison of edar sequence in representative vertebrates. Edar alleles fls dt3Tpl and fls t3R367W positioned above sequence. Sites of splicing defects of fls te370f and fls t0sp213 alleles demarcated with ˆ marker. Yellow, TNFR domain; Grey, transmembrane domain; Green, death domain; Red, polymorphic sites in WIK mapping strain. (0.04 MB DOC) [file pgen.1000206.s002.doc]

**Figure S2**

_ _______ _____________________________**^**__________________ *fls*^te370f^

*D_rerio* 1 ---MGHKGGRTPSFLLHFLLVSSMSTVSAEYSSCGENEFYNHTTSSCQPCPQCQAGQEPYMNCGYGTKDDDYSCVSCPAG
*H_sapiens* 1 ---MAHVGDCTQTPWLPVLVVSLMCSARAEYSNCGENEYYNQTTGLCQECPPCGPGEEPYLSCGYGTKDEDYGCVPCPAE
*M_musculus* 1 ---MAHVGDCKWMSWLPVLVVSLMCSAKAEDSNCGENEYHNQTTGLCQQCPPCRPGEEPYMSCGYGTKDDDYGCVPCPAE
*G_gallus* 1 ---MAHLGECKWTHVFPFLVVSLVYSASAEYSNCGENEYYNQTTGMCHDCPKCEPGEEPYMTCGYGTKDEDYGCIPCPSE
X_laevis 1 ---MNQPGDRTWTHIFRFLMVSLLCSVHADYGNCGENEYYNQTTGMCQSCPECEPGEEPYMSCGYGFKDEDFGCVPCQSG
*G_aculeatus* 1 ANMSERRSQKKSIFLSSLLLCCMF--ASAEYSNCG-YEFFNQTSNSCQACPQCQPGQEPHMNCGYGVKDEDFACVTCPHG
*O_latipes* 1 MKMWKRRGQKKSMFLSSLLVCCMF--ASAEYSSCGEYEFFNQTSNSCQACPQCRPGQEPNMSCGHGMKDEGFACVPCPQG
*F_rubipes* 1 STDMSKRSQKKSVFL-TLFVCWML--VNGEYSSCGEYEFFNQTSNSCQACPQCQPGQEPHMTCGYGMKDEDFACVPCQAG

 _
*D_rerio* 78 KFSKGKYEICRRHKDCDALYRATVLTPGTSDSDAECGHCLPGYYIQENRPQNI-YGMVCHSCQNAPRNIKECMRSTPPAS
*H_sapiens* 78 KFSKGGYQICRRHKDCEGFFRATVLTPGDMENDAECGPCLPGYYMLENRPRNI-YGMVCYSCLLAPPNTKECVGATSGAS
*M_musculus* 78 KFSKGGYQICRRHKDCEGFFRATVLTPGDMENDAECGPCLPGYYMLENRPRNI-YGMVCYSCLLAPPNTKECVGATSGVS
*G_gallus* 78 KFSRGGYQICRRHKDCEGFFRATVLTPGDQENDAECGPCLPGYYMLENRPRNI-YGMVCYSCLLAPPNTKECAGATSGIS
*X_laevis* 78 KYSKGGYQICRRHKDCEGFFRATVLTPGDRENDAECGPCLPGYYMLENRPKSM-YGNVCYSCLSAPPNTKECIGSSAPVS
*G_aculeatus* 78 KYSKGKYEICRRHKDCDALYKATVRVAGTPDSDAECGPCLTGYYMLENRPRNL-YGMVCHSCQNAPRNTKECMLTSRPKE
*O_latipes* 79 KYSKGKYEICRRHKDCNALYKATVREPGTAEKDAECGPCLPGYYMLENRARNL-YAMVCHSCQNAPLNTKECKKTTEAII
*F_rubipes* 78 KYSKGKYEICRRHKDCNALYKATVRVAGTPESDAECGPCLPGYYMLENRPRNQPYGMVCHSCQNAPRNTKECLSTSVPKE

 **___________________________^ __ ^** *fls*^t0sp213^
*D_rerio* 157 GRAPSVSSSST-TIFPQPEK-DPTGQGHLATALIIAMSTIFIMAIAIVMIIMFYILKSKPSGPV-CCSGQLIKAVEAQTN *fls*^tfang^
*H_sapiens* 157 ANFPGTSGSSTLSPFQHAHK-ELSGQGHLATALIIAMSTIFIMAIAIVLIIMFYILKTKPSAPA-CCTSHPGKSVEAQVS
*M_musculus* 157 AHSSSTSGGSTLSPFQHAHK-ELSGQGHLATALIIAMSTIFIMAIAIVLIIMFYIMKTKPSAPA-CCSSPPGKSAEAPAN
*G_gallus* 157 AIFPSTSGTSTFSPYQHAHKADLSGQGHLATALIIAMSTIFIMAIAIVLIIMFYIVKTKPSAQA-CCKSHSVKNVEAQAN
*X_laevis* 157 GSATITSGSSTISPFQHPHKVELSGQGHLATALIIAMSTIFIMAIAIVLIIMFYILKTKPSSQA-CCTG-PSKNVEAQIN
*G_aculeatus* 157 K--PVIDPGST-TVFPHLHK-DSNGQGHLATALIIAMSTIFIMAIAIVLIIMFYILKAKPNSQAACCSGQVVKAVEAQSN
*O_latipes* 158 K--PPINPGST-TVLPHP---GSPGQGHLATALIIAMSTIFIMAIAIVLIIMFYILKAKPNGQA-CCSGQVVKAVEAQTN
*F_rubipes* 158 K--PLIRPGYT-TMFPNPHK-DSTGQGHLATALIIAMSTIFIMAIAIVLIIMFYILKAKPNSQV-CCSGKVVKAVEAQTN


*D_rerio* 234 MQEEKKEAQENVVIFQEKDEFDKLKPSSPKTAKSENDASSENEQLLSRSIDSDEEAAQDKQGA----------------A
*H_sapiens* 235 KDEEKKEAPDNVVMFSEKDEFEKLTATPAKPTKSENDASSENEQLLSRSVDSDEEPAPDKQGS----------------P
*M_musculus* 235 THEEKKEAPDSVVTFPENGEFQKLTATPTKTPKSENDASSENEQLLSRSVDSDEEPAPDKQGS----------------P
*G_gallus* 236 TQEEKKEVQDNVVIFSEKEEFEKLTATPAKAAKSENDASSENERLLSRSMDSDEEAAVDKQGT----------------P
*X_laevis* 235 EQEEKKEVPVE-NIFSEKDEFEKLSETQTKTEKNENDASSENERLLSRSMDSDEEAAIDKQGS----------------A
*G_aculeatus* 233 KQEEKKEIPDKVVIYSEKDEFDKLKAPPQKTVKSENDASSENEQLLSRSIDSDEEAASDKQGSADANNPGLCLVNLGNKP
*O_latipes* 231 KLEDKKDVPDNVVIFPEKEEYDKLKASPQKTVKSENDASSENEQLLSRSIDSDEEPTSDKLRSSEATNHNLCQANVGYKP
*F_rubipes* 233 KQEDKRDVPDNVVIYLEKDEFEKLKAPPPKTVKSENDASSENEQLLSRSMDSDEEAASDKQVAAERGEPSPCLVSLENKP

 T S inN WIK
*D_rerio* 298 DLCLLSLVHLTRDKSC---------------TTNTINNNN--------NHCSRATG-IHSRRKKILDLYTKACSVAEGLS
*H_sapiens* 299 ELCLLSLVHLAREKSA---------------------------------TSNKSAG-IQSRRKKILDVYANVCGVVEGLS
*M_musculus* 299 ELCLLSLVHLAREKSV---------------------------------TSNKSAG-IQSRRKKILDVYANVCGVVEGLS
*G_gallus* 300 ERCLLSLVHLARDKSS---------------------------------TSNKSTG-IQSRRKKILDVYANVCDVAEGLS
*X_laevis* 298 ELCLLSLVHLARDKST---------------------------------TNSKLTG-IHSRRKKILDLYASVCNVAEGLS
*G_aculeatus* 313 DLCLLSLGLLERDRSGNGGPAMSANNIHGANHISSVTNPGGKERRLLKMSHEITTQMLQSRRKKILDLYGRACNVTEGLS
*O_latipes* 311 DLCLLSLGLLDHRVVCNGTPASAGSQANNTQAPNHITSSN-VSSMNAINNNNKTPGMLQSRRKKILDLYARTCHVTEGLS
*F_rubipes* 313 DLCLLSLGLVDRDGGRGRTPAIIACPESSSLSINNVASTNHVSSMNAINSSNKMPGMLQSRRRKILDLYARACNVTEGLS

 W___________________________________________________________F______ *fls*^t3R367W^
*D_rerio* 354 PTELPFDCLERSSRMLSATYSTDKAVVKTWRHLAESFGLKRDEIGGMTDGMQLFDRISTAGYSIPDLLARLLQIERLDAV *fls*^dt3Tpl^
*H_sapiens* 345 PTELPFDCLEKTSRMLSSTYNSEKAVVKTWRHLAESFGLKRDEIGGMTDGMQLFDRISTAGYSIPELLTKLVQIERLDAV
*M_musculus* 345 PTELPFDCLEKTSRMLSSTYNSEKAVVKTWRHLAESFGLKRDEIGGMTDGMQLFDRISTAGYSIPELLTKLVQIERLDAV
*G_gallus* 346 PTELPFDCLEKTSRMLSSTYNTEKAIVKTWRHLAESFGLKRDEIGGMTDGMQLFDRISTAGYSIPELLTKLVQIERLDAV
*X_laevis* 344 PTELPFDCLEKTSRMLSSTYNTEKAIVKTWRHLAESFGLKRDEIGGMTDGLQLFDRISTAGYSIPDLLTRLVQIERLDAV
*G_aculeatus* 393 PTELPFDCLEKASRMLSSSYSSEAAVVKTWRHLAESFGLKRDEIGGMSDGLQLFERVSTAGYSIPDLLTRLVHIERLDAV
*O_latipes* 390 PTELPFDCLEKASRMLSSSYSSDAAVVKTWRHLAESFGLKRDEIGGMSDGLQLFERVSTAGYSIPDLLARLVQIERLDAV
*F_rubipes* 393 PTELPFDCLEKTSRMLSSSYSSEAAVVKTWRHLAESFGLKRDEIGGMSDGLQLFERVSTAGYSIPDLLTRLVQIERLDAV


*D_rerio* 434 ETLCCDILSGTQN------------------CTNPPLSSR--CASV
*H_sapiens* 425 ESLCADILEWAGV--------------------VPPASQP--HAAS
*M_musculus* 425 ESLCADILEWAGV--------------------VPPASPP--PAAS
*G_gallus* 426 ESLCADILEWAQA--------------------MPAPEP---AVTS
*X_laevis* 424 ESLCTDIVEWAQG--------------------TPTLNPP--SSSL
*G_aculeatus* 473 ESLCSDVLGSSET-AAAAGRQGNSSFHSQLVCPSSCLSPSQRCASV
*O_latipes* 470 ESLCADVLGSNEI-VALVGRQSVNSFHSQLVCPSPCTSPSPRCASV
*F_rubipes* 473 ESLCSDVLGVSETAAAAAGRQGIGSFHGQLICASPCSSPSHRCASV
