# Supplementary material for: Zebrafish eda and edar Mutants Reveal Conserved and Ancestral Roles of Ectodysplasin Signaling in Vertebrates
Source: PLoS Genet. 2008 Oct 3;4(10):e1000206. doi: 10.1371/journal.pgen.1000206 (PMC2542418; doi:10.1371/journal.pgen.1000206)
Supplement: Figure S3 — A comparison of eda sequence of representative vertebrates. Blue, transmembrane domain; Green, furin cleavage site; Yellow, TNF domain; asterisk Nkt dtS238X allele; |, deleted residues in alternate spliced form of Eda-2. (0.04 MB DOC) [file pgen.1000206.s003.doc]

**Figure S3**

___________________

*D.rerio* 1 -----MLIDLHAAMEPKREPAKLEERERTQAACECQSQCNNCKIFLSLFILSLSLHLVTL
*H.sapiens* 1 MGYPEVERRELLPAAAPRERGSQGCGCGGAPARAGEGNS--CLLFLGFFGLSLALHLLTL
*M.musculus* 1 MGYPEVERREPLPAAAPRERGSQGCGCRGAPARAGEGNS--CRLFLGFFGLSLALHLLTL
*G.aculeatus* 1 -----MTRDGS-AEDFADKVML---PCTCNKKCRSRSGS---VVFLGLFLLSLSLHAVTL
*O.latipes* 1 -----MACDGSSAVDFPDKVAFRATPCTCGEKCRGQGSS---MVFLGFFLLSLCLHAVTL
*F.rubripes* 1 ----MMARDGSPVEDFPEKVMTGAAPCTCPKRCRSRSGS---VAFLGLFLLSLCLHAVTI
*T.nigroviridis* 1 -----MAHDDPPAEDFPGKAMHGAAPCTCPKKCMTRSGT---KVFLGLFLLSLCLHAVTL

 ____
*D.rerio* 56 FCYLDLRSELKR-EISQKNKD---------EVSSTGPVPHYEATEPVLQS-PDTDHPTIG
*H.sapiens* 59 CCYLELRSELRRERGAESRLGGSGTPGTSGTLSSLGGLDPDSPITSHLGQPSPKQQPLEP
*M.musculus* 59 CCYLELRSELRRERGTESRLGGPGAPGTSGTLSSPGSLDPVGPITRHLGQPSFQQQPLEP
*G.aculeatus* 49 VCYLDLRSEVKR-EIIHQKRD---------TMLTFAGIDLADPAAVLAPG-QPRPDSGIG
*O.latipes* 53 VCYLDLRSEVKREKVLHQRRE---------SVMTLTWS---DPVGVLPPGGYQRQDSRSG
*F.rubripes* 54 VFYMDLRSEVRR-EMIHQKRD---------SILTLAGSDPADP-SVFAPG-PPRLDPGSG
*T.nigroviridis* 53 VFYMDLRSEVRR-ELIHQKRD---------SILTLAGSDPADP-SLFSPG-SQRLDPGSE

 _____
*D.rerio* 105 ----------DQSRWRDEHTRGLER---VIH------RTKRS-------ETNGK--KKGE
*H.sapiens* 119 GEAALHSDSQDGHQMALLNFFFPDEKPYSEEESRRVRRNKRSKSNE------GADGPVKN
*M.musculus* 119 GEDPLPPDSQDRHQMALLNFFFPDEKAYSEEESRRVRRNKRSKSGE------GADGPVKN
*G.aculeatus* 98 ---RGGEGHEEKLLHRNGELHATQDNRGITQ------RAKRSPGKQPETESTGRE-RRKE
*O.latipes* 101 ------ENRQERLQYRNDGFRSTEDHRDITQ------RVKRSSSKETEIESTGKE-KRKD
*F.rubripes* 102 SSRSGGDAHEDKLLHRNNDFHTTEDTRGITQ------RAKRSVGRQPDTESNGRE-KRKE
*T.nigroviridis* 101 -----GDGNEEKLLHRSNDFHTTEDTRAITQ------RAKRSPGKQPDTESHGRE-KRKE


*D.rerio* 137 RKKGKKG-PPGA---PGPPGPPGPQGPPGIPGIPGIPGSNAMGPSGPPGPPGPQGPPGPQ
*H.sapiens* 173 KKKGKKAGPPGPNGPPGPPGPPGPQGPPGIPGIPGIPGTTVMGPPGPPGPPGPQGPPGLQ
*M.musculus* 173 KKKGKKAGPPGPNGPPGPPGPPGPQGPPGIPGIPGIPGTTVMGPPGPPGPPGPQGPPGLQ
*G.aculeatus* 148 KKKGKKRPVPGP---PGPPGPPGPQGPPGIPGIPGIPGSNAVGPAGPPGPPGPQGPPGTP
*O.latipes* 148 KKKGKKRIVPGP---PGPPGPPGPQGPPGIPGIPGIPGSNVVGPAGPPGPPGPQGPPGSQ
*F.rubripes* 155 RKKGKKRSIPGP---PGPPGPPGPQGPPGIPGIPGIPGSNVVGPAGPPGPPGPQGPPGTQ
*T.nigroviridis* 149 RKKGKKRSIPGP---PGPPGPPGPQGPPGIPGIPGIPGSNVVGPVGPPGPPGPQGPPGTQ

 ____________*_________ Nkt
*D.rerio* 193 GPPGPQGPSGGEKGKHREAQPAVVHLQGQETTIQVKEDLSEGVLKNWRMISIHQRVFKMH
*H.sapiens* 233 GPSGAA-----DKAGTRENQPAVVHLQGQGSAIQVKNDLSGGVLNDWSRITMNPKVFKLH
*M.musculus* 233 GPSGAA-----DKTGTRENQPAVVHLQGQGSAIQVKNDLSGGVLNDWSRITMNPKVFKLH
*G.aculeatus* 205 GPAGVP-----DKTKTKEFQPAVVHLQGQETTIQVREDLSEGILRNWKMVSIHHRVFKMH
*O.latipes* 205 GPAGVS-----DKTKTKDFQPAVVHLQGQETTIQVREDLSQGVLKNWKMVSIHHRVFKMH
*F.rubripes* 212 GPAGDP-----DKTKTREFQPAVVHLQGQETTIQVREDLSEGILRNWKMVSIHHRVFKMH
*T.nigroviridis* 206 GPAGDP-----DKTKTREFQAAVVHLQGQETTIQVREDLSEGILRNWKMVSIHHRVFKMH

 ___________________|||______________________________________
*D.rerio* 253 SRSGELEVLLDGTYFIYSQVEVYYLNFTDIASYEVMVDKTPFLRCTRSIETGQRKFNTCY
*H.sapiens* 288 PRSGELEVLVDGTYFIYSQVEVYYINFTDFASYEVVVDEKPFLQCTRSIETGKTNYNTCY
*M.musculus* 288 PRSGELEVLVDGTYFIYSQVEVYYINFTDFASYEVVVDEKPFLQCTRSIETGKTNYNTCY
*G.aculeatus* 260 SRSGELEVLLDGVYFIYSQVEVYYLNFTDIASYEVMVDSNPFLRCTCSIETGQRKFNTCY
*O.latipes* 260 SRTGQLEVLLDGVYFIYSQVEVYYLNFTDIASYDVMVDSHPFLRCTCSIETGQRKFNTCY
*F.rubripes* 267 SRSGELEVLLDGVYFIYSQVEVYYLNFTDIASYEVMVDSNPFLRCTCSLETGQRKFNTCY
*T.nigroviridis* 261 SRSGELEVLLDGVYFIYSQVEVYYLNFTDIASYEVMVDSN-FLRCTCSIETGQRKFNTCY

 _______________________________________________
*D.rerio* 313 TAGVCLLRARQRISIRMVYEDTSISMSNHTTFLGSIRLGDAPSAGHT-
*H.sapiens* 348 TAGVCLLKARQKIAVKMVHADISINMSKHTTFFGAIRLGEAPAS----
*M.musculus* 348 TAGVCLLKARQKIAVKMVHADISINMSKHTTFFGAIRLGEAPAS----
*G.aculeatus* 320 TAGVSLLRAGQRISIRIVYEDTLISMTNHTTFLGSVRLGEAPSAGQN-
*O.latipes* 320 TAGVSPLRAGQKISIRIAHEYTLINMTNHTTFLGSVRLGEAPSAG---
*F.rubripes* 327 TAGVSLLRAGQRISIRIVYEDTLISMTNHTTFLGSVRLGEAPSAGQN-
*T.nigroviridis* 320 TAGVSLLRAGQRISIRIVYEDTLISMTNHTTFLGSVRLGEAPSAGQNR
